# Impact of Laboratory-Adapted Intracellular *Trypanosoma cruzi* Strains on the Activity Profiles of Compounds with Anti-*T. cruzi* Activity

**DOI:** 10.3390/microorganisms11020476

**Published:** 2023-02-14

**Authors:** Melissa L. Sykes, Emily K. Kennedy, Vicky M. Avery

**Affiliations:** 1 Discovery Biology, Centre for Cellular Phenomics, Griffith University, Nathan, QLD 4111, Australia; 2 School of Environment & Science, Griffith University, Nathan, QLD 4111, Australia

**Keywords:** *Trypanosoma cruzi*, parasite strains, drug discovery, compound activity, image-based assay, Chagas disease

## Abstract

Chagas disease is caused by infection with the protozoan parasite, *Trypanosoma cruzi*. The disease causes ~12,000 deaths annually and is one of the world’s 20 neglected tropical diseases, as defined by the World Health Organisation. The drug discovery pipeline for Chagas disease currently has few new clinical candidates, with high attrition rates an ongoing issue. To determine if the *Trypanosoma cruzi* strain utilised to assess in vitro compound activity impacts activity, a comparison of laboratory-adapted *T. cruzi* strains from differing geographical locations was undertaken for a selection of compounds with anti-*T. cruzi* activity. To minimise the possible effect of differences in experimental methodology, the same host cell and multiplicity of infection were utilised. To determine whether the compound exposure time influenced results, activity was determined following exposure for 48 and 72 h of incubation. To ascertain whether replication rates affected outcomes, comparative rates of replication of the *T. cruzi* strains were investigated, using the nucleoside analogue, 5-ethynyl-2′-deoxyuridine. Minimal differences in the in vitro activity of compounds between strains were observed following 48 h incubation, whereas significant differences were observed following 72 h incubation, in particular for the cytochrome P450 inhibitors tested and the cell cycle inhibitor, camptothecin. Thus, the use of panels of laboratory adapted strains in vitro may be dependent on the speed of action that is prioritised. For the identification of fast-acting compounds, an initial shorter duration assay using a single strain may be used. A longer incubation to identify compound activity may alternatively require profiling of compounds against multiple *T. cruzi* strains.

## 1. Introduction

Prior to progression in the Chagas disease drug discovery pipeline, assessment of compound activity across a diverse panel of *Trypanosoma cruzi* strains has previously been recommended [1]. Priority has been given to strains/clones from distinct typing units (DTUs) associated with human infection (TcI, TcII, TcV and TcVI), from a variety of geographical origins [2]. Strain susceptibility testing has been used in drug discovery during lead optimisation [3,4,5]. Strains with varying replication rates may also have the potential to impact compound activity, dependent on the chemical class [1]. The contribution of experimental variables that may influence compound activity, such as the type of host cell, duration of compound exposure and detection methods, must also be factored in for a comparative analysis, as these impact the ability to directly align results not only from different strains, but importantly, different laboratories. Detection methods for intracellular *T. cruzi* vary from Giemsa identification of parasites [6], colorimetric detection of LacZ-transfected parasites [7] and imaging assays to detect intracellular amastigotes [8,9,10,11,12]. Fluorescence imaging of *T. cruzi* infected cells allows visualisation of intracellular parasites over multiple fields, enabling determination of small numbers of infected cells, whether due to low infection rates between strains, or residual parasites following compound exposure.

Few studies utilise different strains of *T. cruzi* in the same host cell for fluorescence-based imaging techniques when comparing compound activity. Moraes et al. have shown that a selection of cytochrome P450 (CYP51) inhibitors have variable IC_50_ values and efficacy when tested across a panel of strains and clones, comprising varying DTUs in U2OS (osteosarcoma) cells [12]. A further study using a *T. cruzi* Y-H10 clone, which exhibits some in vitro resistance to nifurtimox and benznidazole, and the Silvio X10/1 clone (no reported resistance) was undertaken against a panel of host cells: U2OS, THP-1 (macrophage cell line), VERO (epithelial monkey kidney cell line) and L6 myoblasts (rat skeletal cell line) to assess the activity of nifurtimox and benznidazole. [13]. Whilst similar efficacy was observed for benznidazole and nifurtimox against Silvio X10/1 in all host cells, compounds tested against the Y-H10 clone demonstrated variation in both dose response and maximum efficacy [13]. However, the resistance of this *T. cruzi* clone to nifurtimox and benznidazole may also influence outcomes. Benznidazole resistant lines show loss of fitness [14,15] and more studies are needed to establish the extent parasite genetic variability correlates to drug responses, and thus treatment outcome [12]. To determine whether different wild type *T. cruzi* strains influence activity across different chemical classes, we have quantified compound activity and efficacy in various *T. cruzi* laboratory-adapted strains, in the same fibroblast host cell for varying time periods. 

The utilisation of host cells relevant to in vivo models or sites of human infection may help facilitate translation in *T. cruzi* drug discovery. Host cells commonly used in *T. cruzi* compound sensitivity assays include U2OS (human osteosarcoma cell line) [13,16,17], VERO (African green monkey kidney cells) [11,13,18,19], and L6 (rat myoblast) [13,17,20,21,22]. As U2OS is a cancer cell line, up or down regulation of protein expression is likely when compared to its to non-cancerous counterpart [23], which may affect the growth/metabolism of intracellular parasites and hence compound activity. Normal mouse and human cell lines have been utilised in *T. cruzi* in vitro assays in the literature: 3T3 mouse fibroblast cells [3,7,10,24,25], mouse myoblasts [3] and human foreskin fibroblasts (HFF) [19,26]. Fibroblasts have been associated with Chagas disease infection, in human heart tissue [27] and are dispersed throughout connective tissue, with skeletal muscle demonstrated as an area of infection in murine models of Chagas disease [28]. Additionally, parasites interact with/invade epithelial cells and fibroblasts in epithelial and mucous barriers, thus likely facilitating the establishment of chronic infection and may become a long-term reservoir of parasites [29]. More relevant cells to recognised areas of infection in chronic Chagas disease are currently being investigated in vitro, such as human-induced pluripotent stem cell-derived cardiomyocytes [30] and primary cells associated with the gut [31]. It has recently been determined in a mouse model of infection that parasites reside in smooth muscle cells in the colon during the chronic phase of the disease [32]. 

Analysis of how a panel of *T. cruzi* reference strains in the same, normal human or mouse host cell line respond to anti-*T. cruzi* compounds has not previously been reported. To identify differences in *T. cruzi* strain activity in 3T3 mouse fibroblasts, we have assessed the activities of 10 compounds from differing chemical classes on four *T. cruzi* strains from a variety of DTUs. The inclusion of different classes of compounds increased the likelihood of observing differences in *T. cruzi* sensitivity across strains. These compounds represent various chemical classes: the nitro-heterocyclic drugs, benznidazole and nifurtimox; the azoles posaconazole and clotrimazole; ethyl oxo[2-(4-phenyl-1-piperazinyl)-2-(3-pyridinyl)ethyl]aminoacetate (E155-0206) containing a pendent 3-pyridyl motif, camptothecin (a pyranoindolizinoquinoline), ciclopirox olamine (a hydroxypyridone), clemastine fumarate (an ethanolamine) and two compounds from the Medicines for Malaria Pathogen Box collection [33], MMV688958 and MMV688796, a 2-aryl oxazole and a 2,4-substituted furan, respectively. The mode of action (MOA) for some of these compounds in *T. cruzi* include *T. cruzi* CYP51 inhibitors, such as E155-0206 [34], posaconazole [35] and an inhibitor of CYP51 in fungal species, clotrimazole [36]. We have previously identified E155-0206 and clotrimazole as active against *T. cruzi* [10]. We included these representative CYP51 inhibitors (30% of the compounds tested in this study), as this inhibitor class has previously shown significant variation in activity between *T. cruzi* strains [11,12] and appear to be quite prevalent. The mode of action of benznidazole is through multiple targets in the parasite, likely associated with a thiol binding capacity [37] and whilst the activation of nifurtimox is thought to be mediated via type I nitroreductases, the MOA in the parasite is poorly understood [38]. The variable activity of nifurtimox and benznidazole (IC_50_ values) observed between strains was suggested to be due to their relative rates of growth [12]. We also investigated the activity of nifurtimox, benznidazole, and camptothecin; in addition to clemastine fumarate, MMV688958 and MMV688796, which do not inhibit *T. cruzi* CYP51 [34,39]. Camptothecin, a topoisomerase inhibitor in various eukaryotic cells, has been shown to cause cell cycle arrest in *T. cruzi* epimastigotes due to DNA damage [40] and we have demonstrated its activity against *T. cruzi* intracellular amastigotes [10]. Whilst active against *T. cruzi* intracellular amastigotes [10,41], the MOA of ciclopirox olamine associated with *T. cruzi* is unknown; however, it acts as a metal chelator in mammalian cell lines [42,43] and therefore the effect on parasites may be direct, or host cell mediated. 

Differences in the in vitro activity of compounds against of *T. cruzi* strains over time were determined. The *T. cruzi* strains investigated, namely the Y strain (TcII), Tulahuen strain (TcVI), CL strain (TcVI) and G strain (TcI), are utilised in in vivo models, have all demonstrated chronic infection in mice [44,45,46,47] and the CL, Y and Tulahuen strains are commonly used in in vitro drug discovery assays [7,10,11,12,13,17]. The DTUs selected represent distinctly different geographical regions of infection. TCI is representative of the main agent of Chagas disease in the northern part of Latin America and the Amazon Region; TCII is the primary agent of Chagas disease in Atlantic and Central Brazil, TCVI commonly causes Chagas disease in the Gran Chaco/Southern Cone region [1]. Importantly, these are all reference strains [48] available worldwide from commercial suppliers. In addition to representing a variety of DTUs, *T. cruzi* strains with reported different growth/infection rates were evaluated. Tulahuen and Y strain parasites have exhibited differing levels of infection of VERO host cells [11]. Tulahuen and Y strain parasites are described as fast growing in a panel of six strains/clones from DTUs I to VI, investigated by Moraes et al. (2016) [12]. The G strain parasite is used as an example of a low-virulence parasite, causing no detectable parasitaemia in mice, and is only detectable via histological staining [49]. More recent studies have also revealed low levels of the G strain parasite in a murine model of infection, with significantly lower levels in a variety of tissues compared to the CL strain of the parasite [47]. G strain extracellular amastigotes showed higher invasion than those from CL strain parasites in vitro in HELA cells [50,51], although it is also reported that there is lower infection with trypomastigotes for this strain, in comparison to extracellular amastigotes [52]. Whilst the G strain has been reported to grow quickly in vitro [53], this is the first report of the compound sensitivity of this strain. 

To assess the replication of intracellular *T. cruzi* parasites of these strains, we have utilised the nucleoside analogue, 5-ethynyl-2′-deoxyuridine (EdU), as a DNA probe, employing image-based methods that we have previously developed [54]. With this approach, we have determined the replication rates of the four *T. cruzi* strains in this study, to understand if compound activity was influenced by replication rates. Comparative levels of infection, parasite numbers and effect of each strain on the number of host cells over time were also determined and correlated with the sensitivity of strains to compounds. This is the first time that the replication, growth, impact on host cells and sensitivity to compounds of these strains have been investigated head-to-head in one host cell, utilising the same methodology.

## 2. Methods

### 2.1. Compounds

Compounds were prepared in 100% DMSO, at the following concentrations: benznidazole (supplied by EpiChem Pty Ltd., Perth, Australia): 34.7 mM, nifurtimox (Sigma Aldrich, St. Louis, MO, USA): 34.7 mM, ciclopirox olamine: 40 mM, camptothecin 20 mM, clotrimazole 17.5 mM (all purchased from Sigma Aldrich, USA). Ethyl oxo{[2-(4-phenyl-1-piperazinyl)-2-(3-pyridinyl)ethyl]amino} acetate: 20 mM (E155-0206; Chemdiv, San Diego, CA, USA). MMV688958 and MMV688796 were sourced from Enamine (Kyiv, Ukraine) and prepared at 20 mM in 100% DMSO. Compounds were tested in assays in 14 log dilutions to determine IC_50_ values, ranging from 73 µM to 3.7 × 10^−3^ µM for camptothecin, E155-0206, MMV688958 and MMV688796; 64.1 µM to 2.6 µM for clotrimazole; 146 µM to 2.6 µM for ciclopirox olamine and 127 µM to 6.4 × 10^−3^ µM for nifurtimox and benznidazole, to determine IC_50_ values.

### 2.2. Growth of T. cruzi Strains

*T. cruzi* Y strain was provided by Dr Victor Contreras (Universidad de Carabobo, UC, Valencia, Spain), G strain and CL strain were sourced from BEI resources (NR49382 and NR49381, respectively) and Tulahuen strain was kindly provided by Professor Fred Buckner (University of Washington, Seattle, WA, USA). *T. cruzi* strains were provided as epimastigotes and were grown in liver infusion tryptone (LIT) medium, maintained in the log phase of growth and were then grown to the cusp of stationary phase (5–6 days growth), before differentiation to metacyclic trypomastigotes by the use of artificial triatomine urine (TAU3AAG) [55], as we have described previously [10]. Furthermore, 3T3 host cells (ATCC, CCL-92) were sub-cultured in RPMI supplemented with 10% FCS.

Metacyclic trypomastigotes were added to beds of 3T3 host cells and washed with PBS supplemented with Mg^2+^ and Ca^2+^ following 24 or 48 h of incubation. Parasites were sub-cultured once trypomastigotes were released. All cultures were maintained for the same length of time before use in experiments, which in total was 10 weeks post differentiation to metacyclic trypomastigotes, until there were sufficient numbers of trypomastigotes for all strains to utilise for experiments. CL, Y and G strains required a longer period in culture than the Tulahuen strain to obtain sufficient trypomastigotes. The length from inoculation of subcultures to release sufficient trypomastigotes to collect from the supernatant for the assay was 5–6 days for CL strain, 6–7 days for G strain: 5–6 days for Y strain and 5 days for the Tulahuen strain parasite. There was an increase in extracellular amastigotes with the G and Y strain parasites, in comparison to the Tulahuen and CL strain parasites (by visual inspection, results not shown).

### 2.3. T. cruzi Strain Image-Based Assays to Assess Compound Activity

The image-based assays for each *T. cruzi* strain were undertaken as previously described [11] for 48 h incubation and for 72 h incubation [39]. Differing multiplicities of infection were investigated for each parasite and an MOI of 5:1 resulted in the least host cell damage. Briefly, 1 × 10^3^ 3T3 host cells were added to collagen I-coated 384-well plates (PerkinElmer, Waltham, MA, USA) and incubated for 24 h at 5% CO_2_ and 37 °C, before the addition of a MOI of 5:1 parasite: host cells. Parasites that had not infected host cells were washed from wells with PBS supplemented with Mg^2+^ and Ca^2+^ using a Bravo liquid-handling device (Agilent Technologies, Santa Clara, CA, USA) and 5 µL of compounds pre-diluted 1:21 with sterile GenPure™ water (Merck, Rahway, NJ, USA), were added to plates, incubated for 48 or 72 h, and cells were fixed with 4% paraformaldehyde. Staining with Hoechst 33342 identified the nuclei of both the parasite and host cell, and HCS CellMask™ Green (ThermoFisher Scientific, Waltham, MA, USA) defined the cytoplasm of the host cell. Images were acquired on a Phenix confocal, image-based reader (PerkinElmer, Waltham, MA, USA). Fifteen fields of view were imaged for each strain, to ensure the same number of host cells were captured per assay. A Z’-value of >0.5 [56] was obtained for each strain, indicating reproducible assays. Analysis was undertaken to determine the number of infected cells containing ≥5 parasite per cell [10]. Compound activity was calculated utilising 0.4% DMSO as a negative control, 12 µM of nifurtimox as a positive control for parasites and 30 µM puromycin for a positive control for host cells.

### 2.4. Replication of T. cruzi Strains

The replication rates of *T. cruzi* strains were determined with the nucleoside analogue, 5-ethynyl-2′-deoxyuridine (EdU), as we previously described [54]. Briefly, *T. cruzi* infected 3T3 cells were prepared for each strain, at a MOI of 5:1 parasite: host cell, in the same manner as the image-based assays. Infected cells were washed with PBS supplemented with Mg^2+^ and Ca^2+^, before the addition of a final concentration of 1.8 µM EdU. Plates were incubated for 2 h; EdU removed by PBS washing, followed by fixation with PFA, addition of blocking buffer and a click chemistry azide cocktail to identify EdU incorporated into parasite DNA [57]. Wells were co-stained with Hoechst 33342 to identify the nucleus of the parasite and host cell; and CellMask™ Deep Red Plasma membrane stain (ThermoFisher Scientific, Waltham, MA, USA) to identify the cytoplasm of the host cell. Plates were imaged on a Phenix imaging system, utilising 15 fields to align with the number of fields utilised in the image-based assay for each strain. For each parasite, there were 28 replicate wells imaged. The script that was applied to assess replication was as previously described, with some modifications (nucleus area μm^2^ = 60; nuclear roundness >0.4; spot detection: contrast <0.15 and uncorrected spot to region intensity >1.5). The script identifies the number of parasites per well, in infected cells (containing ≥5 parasites per host cell) and the percentage of those cells that are replicating, with a signal in the Alexa488 channel above the background signal.

### 2.5. Calculation of Compound IC_50_ Values, Maximal Achievable Activity (E_max_) and Statistical Analysis

Compound IC_50_ values and the maximum percentage achievable compound activity (E_max_) were determined as previously described [34]. To identify the significance of the differences between IC_50_ values and the percentage activity at the maximum achievable (E_max_) concentrations obtained for compounds against *T. cruzi* strains, an unpaired student’s *t*-test was employed to calculate the *p*-value (GraphPad Prism, Boston, MA, USA), also as previously described [34]. To also identify the significance between the number of parasites per well and the replicative rates for separate strains, an unpaired *t*-test was utilised (GraphPad Prism, Boston, MA, USA).

## 3. Results 

### 3.1. Compound Activity Following 48 h Incubation

**Figure 1:**
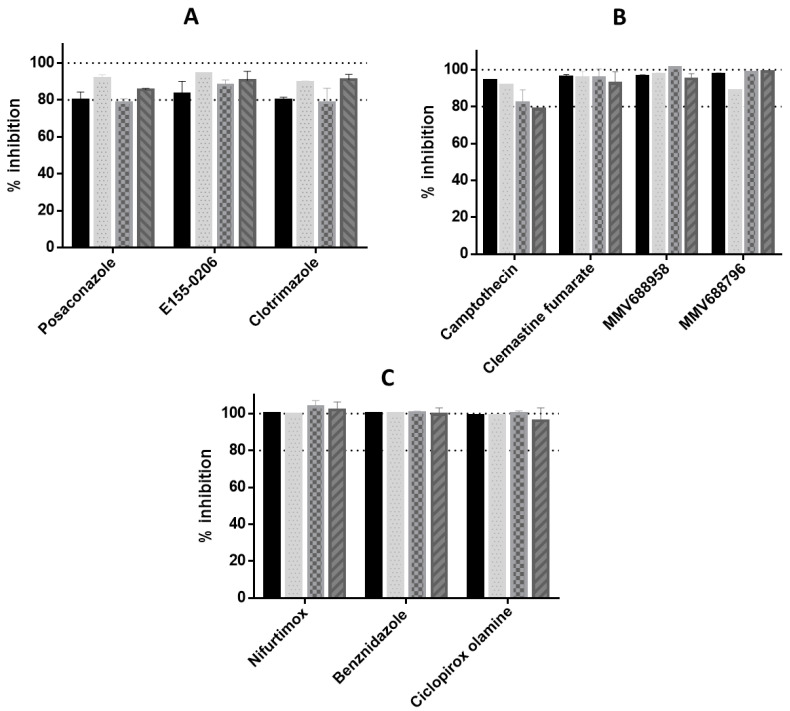
E_max_ values of compounds from differing chemical classes against *T. cruzi* intracellular amastigotes, from Tulahuen, CL, Y and G strain parasites, following 48 h incubation. (**A**) CYP51 inhibitors (**B**) camptothecin, clemastine fumarate and two compounds from the MMV Pathogen Box collection. (**C**) compounds which cleared 100% of parasites from host cells over all strains: the nitroheterocyclic inhibitors benznidazole and nifurtimox; and the antifungal, ciclopirox olamine. Solid bars = Tulahuen strain parasite, dotted bars = CL strain, chequered bars = Y strain, striped bars = G strain. Dashes shown at 80% and 100% activity.

Compounds and drugs with anti-*T. cruzi* activity were compared across a panel of four *T. cruzi* strains following exposure for 48 h. A MOI of 5:1 was utilised for each strain, which caused the least damage to the host cell by parasites, providing reproducible detection of parasites (results not shown). For each strain, the in vitro assay demonstrated a Z’-factor > 0.5, indicating acceptable reproducibility [56]. The E_max_ (maximum efficacy) of the compounds tested were as follows: CYP51 inhibitors (Figure 1A), showed a sub-efficacious effect against all strains; camptothecin and clemastine fumarate did not clear 100% of parasites from host cells (Figure 1B), across all strains. MMV688958 and MMV688796 did not clear parasite from three strains (Figure 1B). The E_max_ activity for MMV688958 was 88%, 95% and 97% for the CL, G and Tulahuen strains, respectively and 99% against Y strain parasites. The E_max_ for MMV688796 was 89% and 96% for CL and Tulahuen strains respectively, and 99% maximum activity against both Y and G strain parasites. Differences >10% in compound efficacy between strains for the two compounds from the MMV Pathogen Box were investigated further to determine if they were significant (*p* < 0.05). The 10% difference in the E_max_ for MMV688956 between the CL and Y, and the CL and G strain parasites were significant (*p* < 0.005). 

The mean E_max_ of the CYP inhibitors investigated was 80 ± 2.3%, 93 ± 1.3%, 83 ± 4.8% and 85 ± 6.3%, for Tulahuen, CL and G strain parasites, respectively, and thus between 80–93% maximum achievable activity. All of the CYP inhibitors showed a significant difference >10% in the E_max_ value to nifurtimox, except for the CL strain, with a mean difference of 93 ± 1.6%, and thus a 7% difference to nifurtimox (100% E_max_). Differences to nifurtimox in the E_max_ of the CYP inhibitors for the CL strain were all significant (*p* < 0.05). 

Minimal differences were observed in the efficacy of CYP51 inhibitors, across all strains (Figure 1A). Differences >10% in the E_max_ between strains were also investigated for these compounds. For posaconazole, a 13% mean difference in the E_max_ between the CL and Y strain parasites and the CL and Tulahuen strains were observed (*p* < 0.05), and a 13% difference in the E_max_ of clotrimazole, between Tulahuen and CL strain parasites (*p* < 0.05). Mean E_max_ differences of 11% for E155-0206 between Tulahuen and CL strain parasites and 10% between Tulahuen and CL strain, with a mean difference in the E_max_ of 15% for clotrimazole between CL and G strains, were reported but were not significant. 

Clemastine fumarate showed <10% difference in the maximum efficacy between strains (Figure 1B). At E_max_ concentrations for each strain clemastine fumarate demonstrated 93–96% inhibition, similar to our previous report illustrating 97% inhibition for the Tulahuen strain [34], which in these studies was 96% against the Tulahuen strain. No difference was observed in the E_max_ for the nitro-heterocyclic drugs used to treat Chagas disease, nifurtimox and benznidazole, and the antifungal ciclopirox olamine (100% efficacy, Figure 1C). Camptothecin was the only compound that showed differences >10% in the E_max_ between strains, with mean differences of 12% 16% and 13% in the inhibition at E_max_ concentrations for Tulahuen and Y strain; Tulahuen and G strain, and the CL and G strain, respectively, with differences significant between Tulahuen and G strains (*p* < 0.05). Camptothecin did not clear parasites from host cells, with 79–94% maximum activity observed for the strains tested (Figure 1B). This was at a concentration that did not influence solubility (7.32 µM), something that was observed at high concentrations (up to 73 µM). There was a significant difference in the mean E_max_ of camptothecin when strains were exposed for 48 h, in comparison to the control nifurtimox, with the exception of the Y strain. There was a difference of 21% maximum inhibition between nifurtimox and camptothecin against the Y strain, with 103 ± 3% and 82 ± 7.1% inhibition shown for these compounds, respectively. This difference was not significant due to variation between the replicate activity of camptothecin against Y strain parasites.

Table 1 shows the IC_50_ value of the compounds tested, against each of the four strains of *T. cruzi* parasites. Compounds that did not clear 100% of parasites from host cells for all strains are indicated (Table 1), specifically the putative and previously identified *T. cruzi* cytochrome P450 (TcCYP51) inhibitors; the cell cycle inhibitor, camptothecin and clemastine fumarate (demonstrated in Figure 1). Differences >2-fold in the IC_50_ value were investigated further to determine the significance of these variations. Of the CYP51 inhibitors, clotrimazole was more active against CL strain in comparison to the other strains, with a six-fold difference in the mean IC_50_ value between CL and G strain (*p* < 0.05), and E155-0206 was more active against the CL strain in comparison to the Y strain with a 2.4-fold difference in the mean IC_50_ value (*p* < 0.05), however a >2-fold, significant variation in activity was not demonstrated by the other CYP51 inhibitors tested. Camptothecin showed a 3.3-fold difference in the IC_50_ value between CL and Y strain parasites (*p* < 0.005) and a 2.2-fold difference between Tulahuen and Y strain parasites (*p =* 0.005). There was no significant difference (*p* < 0.05) observed between the IC_50_ values obtained for the nitroheterocyclic inhibitors and the antifungal compound, ciclopirox olamine against the four parasite strains. 

### 3.2. Compound Activity Calculated Following 72 h Incubation

**Figure 2:**
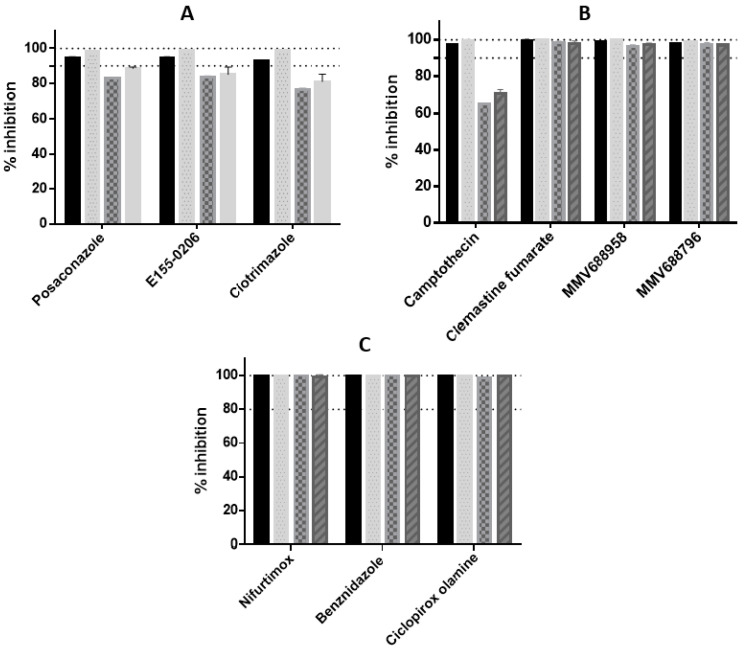
E_max_ values of compounds from differing chemical classes against *T. cruzi* intracellular amastigotes, from Tulahuen, CL, Y and G strain parasites, following 72 h incubation. (**A**) CYP51 inhibitors (**B**) camptothecin, clemastine fumarate and two compounds from the MMV Pathogen Box collection. (**C**) compounds which cleared 100% of parasites from host cells over all strains: the nitroheterocyclic inhibitors benznidazole and nifurtimox; and the antifungal, ciclopirox olamine. Solid bars = Tulahuen strain parasite, dotted bars = CL strain, chequered bars = Y strain, striped bars = G strain. Dashes shown at 90% and 100% activity.

Following 72 h incubation, both intracellular and extracellular parasites were observed in DMSO vehicle (uninhibited) control wells, as we have previously observed [11]. The number of infected cells were determined by identifying parasites only within the cytoplasm of host cells [11], thereby minimising interference by extracellular parasites. Assays for each strain demonstrated a Z’-factor of >0.5, describing a reproducible assay [56]. For each compound, the E_max_ following 48 h incubation also represented the maximal achievable activity following 72 h incubation. Therefore, the same concentration of compound for both incubation times was utilised to enable direct comparison of the E_max_. Compared to the E_max_ following 48 h incubation (Figure 1), an increase in activity was observed for the majority of the CYP inhibitors over time (Figure 2), with a 3–15% increase in activity observed. The greatest increase was observed for the Tulahuen strain parasite, with 15%, 12% and 14% increase in activity for posaconazole, E155-0206 and clotrimazole, respectively. There was a mean increase of 13 ± 2%, for CYP inhibitors, collectively for this strain. The least difference overall for the CYP inhibitors was shown for the Y strain, with a 5% increase in the activity at E_max_ for posaconazole and a decrease in the maximum achievable activity of 7% for clotrimazole and 5% for E155-0206, and thus not a reduction in the number of infected cells. 

An E_max_ of <90% inhibition by CYP inhibitors occurred for the Y and G strain parasites; however, the CYP inhibitors showed >90% activity against the Tulahuen and CL strain parasites. Posaconazole and E155-0206 had an E_max_ of >95% against the Tulahuen strain, and clotrimazole exhibited an E_max_ of 93%. All of the CYP inhibitors showed >95% maximum achievable activity against the CL strain parasite (Figure 2). There was <10% difference in the E_max_ of the three CYP inhibitors for each strain. Therefore, the collective inhibition values at E_max_ for CYP inhibitors were compared between the strains. There was a 13% increase in the E_max_ for the Tulahuen strain compared to the Y strain, and an 18% and 13% increase in the E_max_ for the CL strain compared to the Y and G strains (*p* < 0.0005), respectively. When comparing the E_max_ of the CYP inhibitors to the positive control, nifurtimox, >10%, significant differences were seen for the Y and G strains (*p* < 0.005).

**Figure 3:**
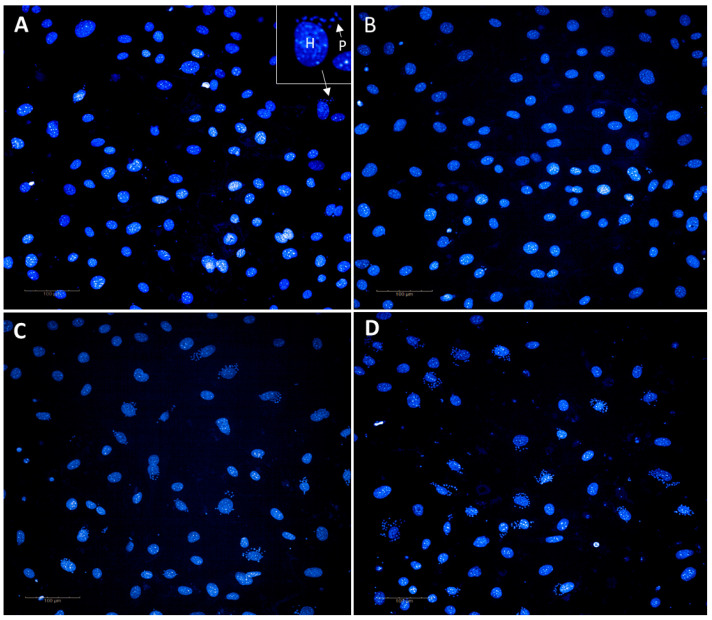
Images of 3T3 infected cells stained with the nuclear dye Hoechst, following treatment with 3.7 µM of the eukaryotic cell cycle inhibitor, camptothecin. Cells infected with: (**A**) Tulahuen strain (**B**) CL strain (**C**) G strain and (**D**) Y strain. The nucleus of parasites and host cells stained with Hoechst are shown in blue, demonstrated in (**A**), inset, where P = parasite nuclei, H = host cell nucleus. A mean of 19.4 ± 2.3% more infected cells was observed for the G and Y strains, in comparison to Tulahuen and CL strains, following treatment. Images taken at 20× magnification using a Phenix (PerkinElmer) imaging system.

There were differences in the E_max_ for camptothecin observed between both the Y and G strains, in comparison to both the Tulahuen and CL strain parasites (Figure 2B). Since the E_max_ was similar for the Tulahuen and CL strains, they were combined to compare differences to other strains. There was a mean difference of 28 ± 1.8% in the E_max_ between the G strain compared to the Tulahuen and CL strain parasites, and a mean difference of 33 ± 1.8% between Y strain compared to the Tulahuen to the G strain parasites. There was ≤50% host cell activity at the E_max_ of camptothecin (at 3.7 µM) of 38%, 32% 47% and 50% activity against 3T3 cells infected with Tulahuen, CL, Y and G strains, respectively (results not shown). This corresponded to a percentage of infected host cells at E_max_ of 3.6 ± 0.49%, 0.30 ± 0.19%, 23 ± 0.74% and 17 ± 0.29% against Tulahuen, CL, Y and G strains, respectively. Images of infected cells for each strain following treatment with 3.7 µM of camptothecin are shown in Figure 3.

Compounds with a >2-fold difference in the IC_50_ value between strains were investigated further to determine if the differences were statistically significant (Table 2). There was a significant difference in the IC_50_ value for camptothecin between the CL and G strain parasites (*p* < 0.005, fold-difference of 2.4). For the CYP inhibitors, there was a significant difference (*p* < 0.05) in the IC_50_ value of clotrimazole against the CL strain compared to Y and Tulahuen strains for E155-0206 with a mean fold difference of 2.1 ± 0.021; and between the CL strain and G strain for clotrimazole (2.3-fold difference, *p* < 0.005). Significant differences in the IC_50_ value were not observed between other strains for the remainder of the CYP inhibitors, where the fold-difference was less than two-fold. The CL strain was more sensitive to the nitroheterocyclic inhibitors (nifurtimox and benznidazole), compared to the other strains (*p* < 0.05, a mean 2.4 ± 0.24-fold difference of collectively), and there was a 2.5-fold difference in the IC_50_ value for ciclopirox olamine against the CL strain, in comparison to the Tulahuen strain (Table 2, *p* < 0.005). 

### 3.3. Number of Infected Cells, Host Cells and Replication of T. cruzi Strains over Time

**Figure 4:**
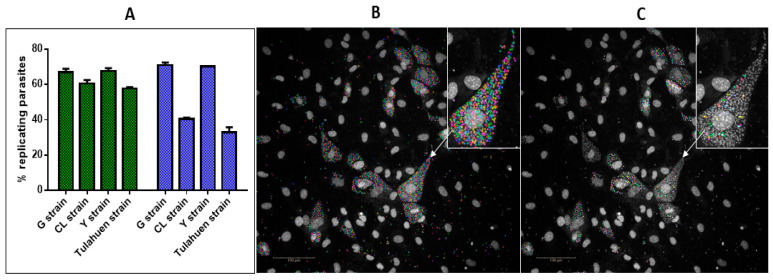
Intracellular replication of four *T. cruzi* strains, detected with the nucleoside analogue 5-ethynyl-2’-deoxyuridine following 48 and 72 h incubation. (**A**) The percentage of replicating intracellular parasites following 48 h (green) and 72 h incubation (blue). (**B**) Hoechst identification of intracellular Tulahuen parasites, following 72 h incubation (**C**) EdU identification of replicating Tulahuen parasite from the same cells as shown in (**A**). In (**B**,**C**) the image inset shows the difference in Hoechst and EdU identified parasites within a cell. Images taken at 20× magnification on a Phenix imaging system (PerkinElmer).

Replication of the *T. cruzi* strains was determined using the nucleoside analogue, 5-ethynyl-2’-deoxyuridine (EdU) [54], following both 48 and 72 h incubation (Figure 4A). After 48 h incubation, less than 10% difference in the percentage of replicating parasites, across all strains, was observed with a mean of 63 ± 4.7% replicating parasites within 3T3 host cells. Not all parasites within host cells were labelled with EdU, which is incorporated into eukaryotic DNA during the S phase of cell division [58]. Following 72 h incubation, a difference in the percentage of dividing parasites detected with EdU was identified between strains. A similar percentage of replicating parasites within host cells infected with the Tulahuen and CL strains (mean of 37 ± 4.6% replicating parasites within host cells) was observed, and also between Y and G strain parasites (71 ± 1% replicating parasites). Between these two sub-groups, there was a significant difference in the number of intracellular replicating parasites (*p* < 0.005). This difference may be due to the presence of trypomastigotes within host cells before egress, rather than a direct comparison of intracellular amastigotes, as *T. cruzi* trypomastigotes do not replicate [59]. Figure 4 shows that the DNA of non-replicating parasites from the Tulahuen strain is more compact within the cell in comparison to host cells containing more replicating parasites (Figure 4B), and additionally, the DNA of these parasites appears to be more elongated. 

**Figure 5:**
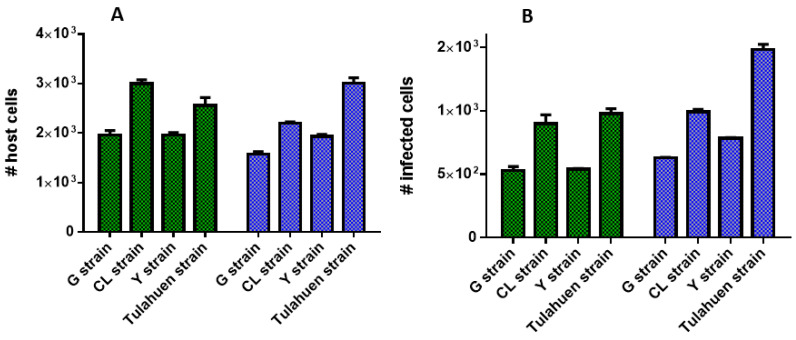
The number of host cells/well (**A**) and number of infected cells/well (**B**) following 48 h (green bars) and 72 h incubation (blue bars) with *T. cruzi* G, CL, Y and G strain parasites, respectively. Data taken from images acquired on a Phenix imaging system (PerkinElmer) and analysed using Harmony 4.8.

A difference in the total number of infected host cells between the Y and G strains, which were similar, and the Tulahuen and CL strains which also showed similarities with each other. After 48 h of incubation, there were 1962 ± 51 and 1965 ± 91 host cells/well following infection with Y and G strain parasites, respectively compared with 2561 ± 160 and 3000 ± 79 host cells/well following infection with Tulahuen and CL parasites, respectively (Figure 5A). After 72 h incubation, similar host cell numbers were observed to the situation following infection with Y and G strain parasites, with 1935 ± 40 and 1582 ± 46 host cells/well, respectively. There were 3008 ± 115 and 2198 ± 30 host cells/well following infection with Tulahuen and CL strains, respectively (Figure 5A). Thus, there were consistently fewer host cells following infection with G and Y strains compared to CL and Tulahuen strains at both time points (Figure 5A). 

Following 48 h incubation, there was a similar level of *T. cruzi* infected 3T3 cells between the G and Y strain, with a mean of 535 ± 8.6 infected cells (Figure 5A). In addition, following 72 h incubation there was a similar number of infected cells between the G and Y strain parasites, with 631 ± 2.8 host cells infected with G strain parasites and 785 ± 5.7 cells infected with Y strain parasites (Figure 5B). Following 72 h incubation, there was a higher number of *T. cruzi* Tulahuen infected cells (1484 ± 41 infected host cells) in comparison to the CL strain (785 ± 5.7 infected host cells). There were also more Tulahuen strain-infected host cells compared to the CL, Y and G strains (*p* < 0.005).

## 4. Discussion

To determine if the *T. cruzi* strain impacted compound activity, a collection of compounds with previously identified activity against the *T. cruzi* Tulahuen strain were tested against a panel of laboratory adapted strains, selected from differing geographical locations. These strains are commonly utilised for in vitro/in vivo drug discovery (Tulahuen, Y and CL parasite strains, or clones of) [10,11,12,24,60] or in vitro/in vivo research (G strain parasites) [47,49,50,51,52,53,61] for Chagas disease. The IC_50_ values and maximum activity (E_max_) of compounds were evaluated using the same host cell to minimise experimental variables that may contribute to differences observed in compound activity between strains. The activity of compounds following 48 and 72 h of incubation was also correlated with parasite replication. Intracellular *T. cruzi* parasites were identified using high content imaging, using well-established, image-based assay and scripts [10,54]. The maximum efficacy (E_max_) and IC_50_ values of compounds were compared between strains, for two time points.

Of the compounds investigated, the CYP51 inhibitors posaconazole, clotrimazole and E155-0206 showed a similar, sub-efficacious E_max_ against the *T. cruzi* strains tested following 48 h of incubation. These compounds had mean maximum achievable activities (E_max_) of 78% ± 2, 93 ± 1%, 83 ± 5% and 79 ± 4% against the Tulahuen, CL, Y and G strains, respectively. After 72 h exposure to CYP inhibitors, an increase in efficacy was observed with the Tulahuen and CL strains, resulting in a mean E_max_ of 94 ± 2% and 99 ± 1% respectively, in comparison to a lower maximum inhibition obtained for Y and G strain parasites, 81 ± 6% and 85 ± 2%, respectively. We have previously reported the lack of in vitro efficacy of CYP inhibitors following 48 h incubation with the Tulahuen *T. cruzi* strain where we defined an E_max_ cut-off <90% activity for 48 h incubation for putative TcCYP inhibitors [34]. Using these criteria, the Tulahuen, Y and G strains tested responded to CYP inhibitors with <90% maximum inhibition following 48 h incubation, whilst the CYP inhibitors inhibited the CL strain with a mean E_max_ of 93 ± 1%. Neither of the CL and Tulahuen strains fulfilled the criteria to define a CYP inhibitor following 72 h of incubation, with 99 ± 1% clearance of infected cells by *T. cruzi* CL strain and 94 ± 2% mean inhibition with the Tulahuen strain. Therefore, a single time point assay of 72 h to assess compound activity may limit detection of putative CYP inhibitors, as not all strains show sub-efficacious activity against *T. cruzi* after this length of incubation. *T. cruzi* CYP inhibitors (TcCYP inhibitors) are not clinically efficacious [62], and are thus commonly deprioritised [5] hence the importance of early identification. 

Other studies have shown variation in the maximum efficacy (E_max_) of CYP inhibitors between *T. cruzi* strains and higher clearance with some strains, where >48 h of incubation was utilised to assess compound activity. Whilst a time point prior to 48 h was not tested, >90% efficacy of the majority of the strains tested was shown following 120 h incubation, with the CYP inhibitor posaconazole, where a panel of *T. cruzi* strains were used to infected VERO cells [12]. Differences were however observed in the efficacy between strains. Posaconazole was efficacious (100% inhibition) against *T. cruzi* M6241 and Y strain parasites, and >90% efficacious against Tulahuen and Silvio X10/7 strains, although 65% maximum activity was demonstrated against the PAH179 strain [12]. Moraes et al. reported 93% efficacy following exposure of Tulahuen strain to posaconazole for 96 h, compared to 57% efficacy for CL Brener, and 84% for Y strain. The host cell in this case was U2OS cells, with image-based detection of the ratio of infected cells, stained with DRAQ5. CL Brener also exhibited a low efficacy following exposure to the CYP51 inhibitors ravuconazole, EPL-BS967 and EPL-BS1246, with a mean E_max_ of 52% [12]. The CL Brener clone utilised (a clone of CL strain) may have some resistance to the *T. cruzi* CYP51 inhibitors tested. However, this was not determined. CYP inhibitors in our study inhibited 99% of the non-clonal CL strain following 72 h of incubation. In vitro assay parameters may have also attributed to this difference in the E_max_ observed. In the Moraes study, following infection of host cells, parasites were not washed off, whereas in our study, parasites that had not infected host cells were washed out of wells following 24 h incubation [10]. Since the CL Brener clone was added to host cells at a higher MOI than either the Y or Tulahuen strains (20:1, 4:1 and 15:1, respectively), this may influence compound activity by the presence of extracellular amastigotes. Further studies have modified the image-based assay in the Moraes publication to incubate host cells for an additional 24 h following parasite addition and before compound addition (48 h in total), to reduce extracellular parasites [17]. External parasites may influence the efficacy of inhibitors, particularly where compounds are known to interact with cellular membranes and their related components [17], such as CYP inhibitors [63]. In the modified assay reported by Yang et al. [17] the efficacy of the CYP inhibitors tested (the azole antifungals posaconazole and ravuconazole, fenarimol and two analogues of fenarimol: EPL-BS967 and EPL-BS1246) increased, in some cases around 50% compared to the original format. The Y strain parasite was only utilised in this study, with both 48 and 72 h incubation periods. Additionally, to calculate compound activity, the number of parasites per well was used, whereas the original format reported in the Moraes publication utilised the infection ratio (percentage of host cells infected with parasite) to calculate compound activity. Using the infection ratio, no activity was reported following 48 h incubation with the CYP inhibitors tested. However, activity was demonstrated by these compounds when activity was calculated based on the number of parasites. The reasoning suggested for this lack of activity was due to the calculation of activity using the ratio of infected cells, as infected cells with few parasites remaining are counted even with decreases of overall parasite numbers, thus an effective compound could be quantified as inactive [17]. However, we show that this is not the case using number of infected cells to calculate compound activity in our model, where inhibitory activity was exhibited for each of the CYP inhibitors tested following 48 h exposure. It may be that utilising the ratio of non-infected cells to calculate compound activity [17] rather than the number of infected cells causes a reduction in sensitivity due to low parasite infection rates in relation to host cells, although this comparison was not made. Additionally, the assay format reported by Yang et al. used a 72 h assay (48 h of incubation with parasite), and non-infected parasites are not washed off; therefore, extracellular parasites could potentially interfere with compound activity. With the assays in our study, trypomastigotes were washed off before compound addition [10], and therefore external parasites are less likely to affect compound activity, and there is minimal parasite egress in the 48 h assay format. The number of infected cells was used to assess compound activity, where the minimal number of detectable parasites (≥5 parasites per cell) was used to define an infected cell, considering that clearance from individual host cells would be required during treatment. We found a mean 10% increase in E_max_ of CYP inhibitors across *T. cruzi* strains when using the number of parasites per well (results not shown) to calculate compound activity. Whilst experimental variables influence compound activity between studies, we have aimed to reduce variables that may interfere with the comparison of compound activity between strains in our methods. 

Following 48 h incubation, there were significant differences in IC_50_ values between the CL strain and either the Y or G strain, for three compounds. There was a six-fold increase in the activity of clotrimazole against CL strain compared to G strain parasites and a three-fold increase in the IC_50_ value of camptothecin against the CL strain compared to Y strain. Following 72 h of incubation, further differences were observed between CL and other strains, consisting of an increase in the activity of E155-0206 compared to the Tulahuen and Y strains (two-fold decrease in the IC_50_ value); clotrimazole showed 2.3-fold more activity against the CL strain compared to the G strain and camptothecin a 2.4-fold increase in activity compared to the G strain. There was also an increase in activity (decrease in IC_50_ value) by the nitroheterocyclic drugs benznidazole and nifurtimox against the CL strain compared to the other strains, with a 2.2–2.8-fold increase in the IC_50_ value of benznidazole and a 2.1–2.7-fold-increase for nifurtimox. Moraes et al. also found the CL Brener clone to be more sensitive to benznidazole than the Y strain with a 2.5-fold reduction in the IC_50_ value following 96 h of incubation. The nitroheterocyclic drugs were also less active against the Y strain compared to the Tulahuen strain, with a 3.8-5.8-fold reduction in the IC_50_ values, and additionally less active against the majority of the panel of parasites investigated [12]. Other studies have also shown a decrease in the susceptibility of the Y strain to benznidazole and nifurtimox, in comparison to other strains. Maclean et al. (2018) reported a reduction in *T. cruzi* Y strain sensitivity to benznidazole when compared to a panel of strains (TcI, Silvio X10/7, Y strain, M6241, ERA, PAH179 and Tulahuen) following exposure for 120 h, where the number of parasites/well was utilised to calculate compound activity [11]. In this method, pre-infected host cells were added to 384-well plates containing the compound. Benznidazole was less active against Y strain parasites than the other strains tested, with 5–13-fold less activity. In their study, infection with the Tulahuen strain resulted in 23% infected cells and Y strain infection resulted in 11% infected cells; which may be due to the differences in cycling times (time until egress) observed between these strains [11]. We also observed fewer intracellular Y strain parasites in comparison to the Tulahuen strain, with 28% infected cells for Y strain and 38% infected cells with Tulahuen strains, following 48 h of incubation post-parasite wash off; and after 72 h incubation, there were 41% and 49% infected cells for the Y and Tulahuen strains, respectively. We did not however observe a reduction in Y strain sensitivity to the nitroheterocyclic drugs compared to the other strains tested. *T. cruzi* Y strain sensitivity to benznidazole has been reported with other assay formats/life cycle forms of *T. cruzi*, although there appear to be variable levels of resistance to these drugs reported for the Y strain in the literature. Whilst not the clinically relevant life cycle stage of *T. cruzi*, studies using epimastigotes showed no difference in the sensitivity of strains to benznidazole regardless of their in vivo sensitivity [64]. Alternately, some studies showing that benznidazole has a similar effect against susceptible and partially resistant *T. cruzi* strains in vivo, also stated that the Y strain was partially resistant and CL strain was sensitive [65]. Other studies have referred to Y strain parasites as resistant [66] in vivo. It may be that this strain demonstrates more pronounced resistance in vivo or variations in methodology, or the genotypes found among different laboratory strains, could influence the sensitivity of Y strain parasites [12]. Here, we show that laboratory adapted Y strain parasites did not demonstrate in vitro resistance to benznidazole or nifurtimox and there were no differences in the efficacy of the nitrohetrocyclic inhibitors following 48 and 72 h of incubation, for any of the strains tested. 

Camptothecin was the only compound in this study that showed variation in efficacy between *T. cruzi* strains, for both time points investigated. After 48 h of incubation, most differences in E_max_ were just above 10%, whereas greater differences were seen following 72 h, with the lowest efficacy displayed against Y strain parasites, with a mean E_max_ of 65% and the highest for Tulahuen strain, with an E_max_ of 97%, a difference of 32%. As camptothecin is an inhibitor of *T. cruzi* replication in epimastigotes, mediated by DNA damage [40] and is a topoisomerase inhibitor in other organisms [67], this may be indicative of the MOA in the intracellular amastigote life cycle form. Topoisomerase inhibitors have been identified with activity against *T. cruzi*, although reports on parasite specific topoisomerase I inhibitors are lacking, and the majority of these inhibitors have been tested against epimastigotes [68,69,70]. We initially reported the activity of this camptothecin against the *T. cruzi* Tulahuen strain following 48 h of incubation [10], with this report the first describing activity for 72 h and against different *T. cruzi* strains. Although host cell activity was also observed with camptothecin after 72 h of incubation, a plateau of maximum activity was not reached, with ≤50% activity against 3T3 host cells, at a final assay concentration of 7.3 µM. Camptothecin and its analogues have demonstrated anti-cancer properties and currently used both alone and in combination to treat a variety of different cancers. As 3T3 fibroblasts are contact inhibited and thus have limited replication, the efficacy of camptothecin may be limited, which may be further impacted as *T. cruzi* infection has been demonstrated to inhibit the proliferation of 3T3 fibroblasts [29]. Previous studies indicate survival of *T. cruzi* Y strain epimastigotes following incubation with 1 µM camptothecin, for 72 h. Flow cytometry analysis of treated epimastigotes, stained with Annexin V, suggest that parasites undergo cell cycle arrest and enter early apoptosis, in addition to loss of mitochondrial membrane potential. Cell cycle arrest and apoptosis were reversible, suggesting that camptothecin treated parasites did not enter late apoptosis [40], although this would need to be confirmed for the amastigote life cycle stage of the parasite.

To determine if the replication rates contribute to the differences in efficacy and activity of compounds following 72 h incubation with different strains, we labelled replicating parasites with EdU [54]. Maclean et al. utilised EdU to show there were less replicating intracellular *T. cruzi* PAH179 strain parasites in comparison to Silvio X10/7 following 120 h incubation, which may be associated with the lower efficacy of posaconazole observed in their studies against PAH179 [11]. We found no differences in the (percentage) replication of Tulahuen, Y, CL and G strain following 48 h incubation, although after 72 h, there was a reduction in the percentage of replicating CL and Tulahuen strain parasites, by 32% and 42%, respectively. However, it appeared that these two strains had more intracellular trypomastigotes within host cells at this time point. These are not the replicating form of the parasite, and thus not an indication of a more slowly growing parasite strain in our studies. Intracellular DNA replication and differentiation of *T. cruzi* is asynchronous within individual host cells in vivo at all stages of infection [71]. Additionally, following 72 h incubation we observed extracellular amastigotes in untreated wells for all *T. cruzi* strains tested, which may influence the ability of slower-acting compounds such as the CYP inhibitors and camptothecin to inhibit intracellular parasites. We observed during the in vitro subculture of Y and G strain parasites, that there was more egress of amastigotes from host cells for these strains than the Tulahuen and CL strain (results not shown). Extracellular amastigotes are thought to be generated by premature lysis of infected host cells, or by differentiation of extracellular trypomastigotes [72]. As CYP51 inhibitors and camptothecin were less efficacious against Y and G strain parasites, in addition to the potential effect of host cells as described, extracellular amastigotes could reduce compound activity/ efficacy [17]. Where *T. cruzi* image-based assays are reported, the presence of extracellular parasites is not often described. We found, following 48 h of incubation (after washing off non-infected parasites), that there were minimal extracellular parasites observed. Therefore, additional experimental variables that can influence compound activity and detection of replication following a longer incubation should be considered. 

The effect of *T. cruzi* strains on host cell health could additionally influence the differences in compound activity observed following 72 h of incubation. It has been shown that some strains have apoptotic effects on host cells. Infection with *T. cruzi* DM28c strain was shown to reduce the number of 3T3 fibroblasts by altering fibroblast proliferation, triggering a cellular stress response and induction of a senescent-like phenotype [29]. Infection of a panel of non-immune host cells, consisting of epithelial cells, fibroblasts and smooth muscle cells with Y strain parasites, also caused upregulation of apoptotic regulators and impediment of cell cycle [73]. Accumulation of ROS in host cells, as observed in fibroblasts following infection with *T. cruzi* [29], may favour the growth/survival of *T. cruzi* [74]. We found a mean 16% reduction in host cell numbers with the Y and G strain parasites compared to Tulahuen and CL strain parasites, for both time points investigated. This potentially apoptotic effect may contribute to the survival of small numbers of parasites following exposure to CYP inhibitors/camptothecin, for these strains. 

From this study, we show that there is a difference in host cell and parasite survival between strains; however, significant differences in parasite survival were observed following 72 h of incubation. Minimal differences in the activity of compounds tested against different *T. cruzi* strains were observed following 48 h of incubation. Aside from the CYP inhibitors and camptothecin, the majority of compounds were fast-acting and reduced intracellular parasite populations by >90%, following 48 h of incubation. A slightly lower efficacy was observed for the MMV Pathogen Box compounds MMV688958 and MMV688796 [33] which showed 88% and 89% maximum activity respectively against the CL strain, and thus may be considered borderline in terms of potential CYP activity [34]. However, we have identified that these compounds are not CYP inhibitors [39] and future correlation with in vivo activity would be beneficial. Clemastine fumarate had a E_max_ that ranged from 92–96% against *T. cruzi* strains after 48 h of incubation, and whilst the majority of infected cells were cleared by this compound, and all parasites following 72 h of incubation, clemastine fumarate did not inhibit parasites in a *T. cruzi* in vivo model of infection using the Tulahuen strain [60]. Recently, clemastine fumarate was found to suppress in vivo leishmania infection in both in vitro and in vivo models of infection of cutaneous leishmaniasis, in addition to targeting inositol phosphorylceramide synthase in the parasite, a component of the sphingolipid biochemical pathway [75]. 

Following 72 h of incubation, differences in activity/efficacy between strains were observed, particularly for the CYP inhibitors and the cell cycle inhibitor, camptothecin. If we consider these differences to be based on either host- or parasite-mediated effects, slow-acting compounds may require confirmation of activity against multiple strains in vitro. The necessity of strain profiling may depend upon the compound speed of action that is of priority, and thus the time of incubation. An initial shorter assay, potentially against one strain, can be utilised to prioritise fast-acting compounds and additionally have less interference from extracellular parasites. Fast-acting compounds are a priority for many screening campaigns due to the poor clinical efficacy of slow-acting compounds [5]. However, if both slow- and fast-acting compounds are of interest, a longer initial incubation of 72 h or greater could precede time-to-kill studies, with multiple strains. This would enable filtering of potential CYP inhibitors, and identification of other slow-acting compounds with differing modes of action. Ideally, in vitro activity should be correlated with in vivo efficacy across these strains to confirm if the differences between strains translate.

## Figures and Tables

**Table 1:** IC_50_ values of compounds with known activity against *T. cruzi* following 48 h incubation, against differing parasite strains: the cytochrome P450 inhibitors posaconazole, E155-0206 and clotrimazole; the cell cycle inhibitor camptothecin; clemastine fumarate and compounds from the MMV Pathogen Box collection (unknown MOA against *T. cruzi*) and drugs used to treat Chagas disease (nifurtimox and benznidazole). ^1^ Indicates compounds that did not clear 100% of parasites from host cells, across all parasite strains, with ≤95% maximum achievable activity (E_max_). ^2^ Significance = a significant difference in the IC_50_ value of compounds between strains (*p* < 0.05), shown where the IC_50_ for the strain is two-fold less than another.

	IC_50_ Value against *T. cruzi* Strains (µM)			
Compound	Tulahuen	Y	G	CL
Posaconazole ^1^	1.6 × 10^−3^ ± 9.6 × 10^−4^	0.71 × 10^−3^ ± 1.2 × 10^−4^	0.94 × 10^−3^ ± 8.5 × 10^−4^	0.74 × 10^−3^ ± 9.5 × 10^−5^
E155-0206 ^1^	1.03 ± 0.35	1.2 ± 0.15	1.4 ± 0.79	0.503 ± 0.045
Clotrimazole ^1^	0.072 ± 0.050	0.11 ± 0.041	0.14 ± 0.037	0.023 ± 0.0021
Clemastine Fumarate	0.65 ± 0.046	0.66 ± 0.015	0.44 ± 0.19	0.36 ± 0.13
Camptothecin ^1^	0.205 ± 0.012	0.46 ± 0.029	0.28 ± 0.13	0.14 ± 0.01
Ciclopirox olamine	2.9 ± 0.63	1.9 ± 1.1	1.5 ± 0.22	1.8 ± 0.27
Nifurtimox	1.6 ± 0.34	1.7 ± 0.23	2.5 ± 0.73	0.81 ± 0.040
Benznidazole	8.01 ± 0.25	6.6 ± 0.73	4.3 ± 1.2	5.5 ± 0.78
MMV688958	0.61 ± 0.027	0.56 ± 0.025	0.65 ± 0.037	0.61 ± 0.017
MMV688796	0.57 ± 0.042	0.51 ± 0.018	0.61 ± 0.15	0.81 ± 0.16
Significance ^2^	TULA/Y (CPT)	See CL/TULA	See CL	CL/G (CTZ) CL/Y (E155-0206, CPT)

**Table 2:** IC_50_ values of compounds with known activity against *T. cruzi* determined after 72 h incubation with different strains: the cytochrome P450 inhibitors posaconazole, E155-0206 and clotrimazole; the cell cycle inhibitor camptothecin; clemastine fumarate and two compounds from the MMV Pathogen Box collection (unknown MOA against *T. cruzi*) and the drugs used to treat Chagas disease (nifurtimox and benznidazole). ^1^ Indicates compounds that did not clear 100% of parasites from host cells, across all parasite strains, with ≤95% maximum achievable activity (E_max_). ^2^ There was a 30–50% reduction in host cell numbers at the E_max_, for camptothecin. ^3^ Significance = a significant difference in the IC_50_ value of compounds between strains (*p* < 0.05), shown where the IC_50_ for the strain is 2-fold less than another.

	IC_50_ Value against *T. cruzi* Strains (µM)			
Compound	Tulahuen	Y	G	CL
Posaconazole	0.97 × 10^−3^ ± 3.9 × 10^−5^	1.6 × 10^−3^ ± 2.02 × 10^−4^	2.03 × 10^−3^ ± 5.02 × 10^−4^	0.89 × 10^−3^ ± 0.89 × 10^−4^
E155-0206	0.70 ± 0.032	0.67 ± 0.048	0.53 ± 0.043	0.33 ± 0.014
Clotrimazole	0.057 ± 0.013	0.069 ± 0.019	0.077 ± 0.042	0.033 ± 0.0064
Clemastine fumarate	0.99 ± 0.22	0.72 ± 0.14	0.66 ± 0.082	0.49 ± 0.048
Ciclopirox olamine	2.03 ± 0.13	0.89 ± 0.081	0.60 ± 0.013	0.81 ± 0.013
Camptothecin ^1,2^	0.16 ± 0.015	0.37 ± 0.15	0.22 ± 0.002	0.093 ± 0.004
Nifurtimox	1.3 ± 0.15	1.3 ± 0.12	1.0 ± 0.20	0.46 ± 0.020
Benznidazole	8.3 ± 1.04	6.0 ± 0.043	6.4 ± 0.91	3.04 ± 0.17
MMV688958	0.80 ± 0.12	0.69 ± 0.057	0.69 ± 0.095	0.62 ± 0.025
MMV688796	1.6 ± 0.053	1.2 ± 0.022	1.05 ± 0.0701	1.05 ± 0.0067
Significance ^3^	See CL	See CL	See CL	CL/Y, TULA (E155-0206) CL/G (CTZ) CL/TULA (CPX) CL/all (NFX) CL/all (BZ) CL/G (CPT)
